# Supplementary material for: Nonequilibrium noise as a probe of pair-tunneling transport in the BCS–BEC crossover
Source: PNAS Nexus. 2023 Feb 9;2(3):pgad045. doi: 10.1093/pnasnexus/pgad045 (PMC9991511; doi:10.1093/pnasnexus/pgad045)
Supplement: pgad045_Supplementary_Data [file pgad045_supplementary_data.zip › PNASNEXUS-PNASNEXUS-2022-00994-T-s03.pdf]

Supporting Information for

Nonequilibrium noise as a probe of pair-tunneling transport in the BCS–BEC crossover

Hiroyuki Tajima, Daigo Oue, Mamoru Matsuo, and Takeo Kato

Hiroyuki Tajima
E-mail: hiroyuki.tajima@phys.s.u-tokyo.ac.jp

This PDF file includes:

Supporting text
Figs. S1 to S2
SI References

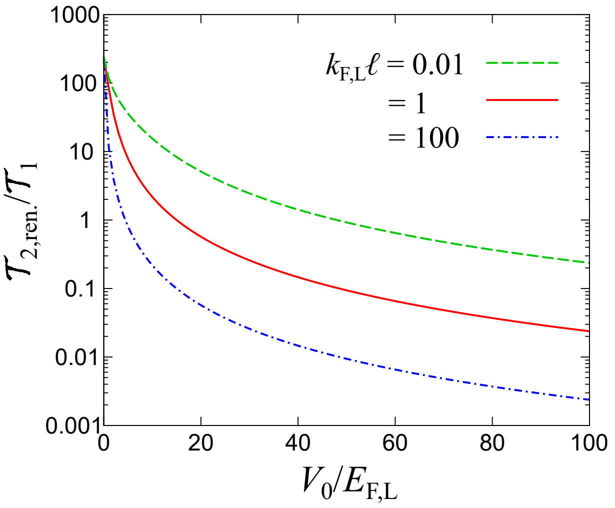

Fig. S1. The dimensionless ratio between $\mathcal{T}_{2,\mathrm{ren.}}$ and $\mathcal{T}_1$ given by Eq. (5), where we used $k_{\mathrm{F,L}} r_{\mathrm{eff}} = 10^{-2}$. V_0 and ℓ are defined through the delta-function form of the potential barrier $V(x) = V_0\delta(x/\ell)$.

Supporting Information Text

Pair-tunneling coupling

Following Ref. (1), we obtain the renormalized pair-tunneling coupling as

$$\mathcal{T}_{2,\mathrm{ren.}} \equiv \frac{\Lambda^2 k_{\mathrm{F,L}}}{3\sqrt{2}\pi^2}\mathcal{T}_2 \simeq \frac{\Lambda^2 k_{\mathrm{F,L}}}{3\sqrt{2}\pi^2} 2|g|\mathrm{Re}[B_{0,\uparrow}B_{0,\downarrow}] \tag{1}$$

where $B_{0,\sigma}$ is the amplitude of the transmitted wave with respect to the potential barrier. Since we consider the spin-balanced system, we take $B_{0,\uparrow} = B_{0,\downarrow} \equiv B_0$. Near unitarity ($a^{-1} \simeq 0$), g can be rewritten as $g = \frac{4\pi a}{m}\frac{1}{1-\frac{4\pi a}{m}\frac{m\Lambda}{2\pi^2}} \simeq -\frac{2\pi^2}{m\Lambda}$. While g is negative, $\mathcal{T}_{2,\mathrm{ren.}}$ can be taken to be positive by the appropriate gauge transformation. In the case of the delta potential barrier $V(x) = V_0\delta(x/\ell)$ being perpendicular to the x axis (ℓ is the typical length scale of the tunneling region, e.g., the width of the actual potential barrier), which is given by a constant $V(\boldsymbol{k}) = V_0$ in the momentum space, we find (2)

$$\mathrm{Re}[B_0^2] \simeq T_{\mathrm{trans.}} = \frac{1}{1+\frac{mV_0^2\ell^2}{2E_{\mathrm{F,L}}}}, \tag{2}$$

where $T_{\mathrm{trans.}}$ is the transmission coefficient. For simplicity, we take $E_{\mathrm{F,L}}$ for the energy of the incident particle and the transverse motion along the barrier is neglected. Combining them, we get

$$\mathcal{T}_{2,\mathrm{ren.}} \simeq \frac{4\Lambda k_{\mathrm{F,L}}}{3\sqrt{2}m}\frac{1}{1+\frac{mV_0^2\ell^2}{2E_{\mathrm{F,L}}}}. \tag{3}$$

In turn, we obtain the quasiparticle-tunneling coupling $\mathcal{T}_1$ as

$$\mathcal{T}_1 \simeq B_0(E_{\mathrm{F,L}}+V_0) \equiv \frac{E_{\mathrm{F,L}}+V_0}{\sqrt{1+\frac{mV_0^2\ell^2}{2E_{\mathrm{F,L}}}}}, \tag{4}$$

where we ignore the higher order term involving the reflection amplitude. Note that the Hartree term gN_{L} is negligible compared to the other term for the present short-range interaction. In this way, we obtain

$$\frac{\mathcal{T}_{2,\mathrm{ren.}}}{\mathcal{T}_1} \simeq \frac{4}{3\sqrt{2}}\frac{\Lambda k_{\mathrm{F,L}}}{m(E_{\mathrm{F,L}}+V_0)}\frac{1}{\sqrt{1+\frac{mV_0^2\ell^2}{2E_{\mathrm{F,L}}}}} \equiv \frac{8}{3\sqrt{2}}\frac{\Lambda/k_{\mathrm{F,L}}}{\left(1+\frac{V_0}{E_{\mathrm{F,L}}}\right)\sqrt{1+\left(\frac{V_0}{E_{\mathrm{F,L}}}\right)^2(k_{\mathrm{F,L}}\ell)^2}}. \tag{5}$$

While we use the contact-type interaction with the cutoff regularization, the cutoff Λ can be associated with the effective range r_{eff} as $r_{\mathrm{eff}} = \frac{4}{\pi\Lambda}$ (and moreover the interaction range $r_{\mathrm{int.}}$) (3). In cold atom experiments, the typical interaction range is approximately given by $|k_{\mathrm{F,L}} r_{\mathrm{int.}}| \simeq 10^{-2}$ (4, 5).

Figure S1 shows $\mathcal{T}_{2,\mathrm{ren.}}/\mathcal{T}_1$ in Eq. Eq. (5) as a function of $V_0/E_{\mathrm{F,L}}$ at different $k_{\mathrm{F,L}}\ell$. One can see that the ratio can be tuned by changing V_0.

Schwinger-Keldysh approach for current and noise

We start from the current operator given by

$$\hat{I} = \hat{I}_{\rm qp} + \hat{I}_{\rm pair}, \tag{6}$$

$$\hat{I}_{\rm qp} = i \sum_{\boldsymbol{p},\boldsymbol{k},\sigma} t_{\boldsymbol{k},\boldsymbol{p}} \left[c^{\dagger}_{\boldsymbol{k},\sigma,\rm L} c_{\boldsymbol{p},\sigma,\rm R} - c^{\dagger}_{\boldsymbol{p},\sigma,\rm R} c_{\boldsymbol{k},\sigma,\rm L} \right], \tag{7}$$

$$\hat{I}_{\rm pair} = 2i \sum_{\boldsymbol{q},\boldsymbol{q}'} w_{\boldsymbol{q},\boldsymbol{q}'} \left[P^{\dagger}_{\boldsymbol{q},\rm L} P_{\boldsymbol{q}',\rm R} - P^{\dagger}_{\boldsymbol{q}',\rm R} P_{\boldsymbol{q},\rm L} \right], \tag{8}$$

where $\hat{I}_{\rm qp}$ and $\hat{I}_{\rm pair}$ are operators for quasiparticle and pair currents, respectively. Truncating the higher-order contributions with respect to the tunneling Hamiltonians [i.e.,$O(H_{\rm 1T}^3)$, $O(H_{\rm 2T}^3)$], we can evaluate their expectation values, $I_{\rm qp}(t_1,t_2) = \langle \Psi(t_1)|\hat{I}_{\rm qp}|\Psi(t_2)\rangle$ and $I_{\rm pair}(t_1,t_2) = \langle \Psi(t_1)|\hat{I}_{\rm pair}|\Psi(t_2)\rangle$, for the different times t_1 and t_2, where $|\Psi(t)\rangle$ is the state-vector of the steady state. First, the quasiparticle contribution reads

$$I_{\rm qp}(t_1,t_2) = -2\int_C dt' \sum_{\boldsymbol{p},\boldsymbol{k},\sigma} |t_{\boldsymbol{k},\boldsymbol{p}}|^2 \,{\rm Re}\left[\langle T_C c_{\boldsymbol{k},\sigma,\rm R}(t_2) c^{\dagger}_{\boldsymbol{k},\sigma,\rm R}(t')\rangle \langle T_C c_{\boldsymbol{p},\sigma,\rm L}(t') c^{\dagger}_{\boldsymbol{p},\sigma,\rm L}(t_1)\rangle \right], \tag{9}$$

where C denotes the Keldysh contour. Note that while the right hand side of Eq. (9) depends only on $t_1 - t_2$ in considering the steady state. Using the Green's functions, we rewrite $I_{\rm qp}(t_1,t_2)$ as

$$I_{\rm qp}(t_1,t_2) = 2\int_{-\infty}^{\infty} dt' \sum_{\boldsymbol{p},\boldsymbol{k},\sigma} |t_{\boldsymbol{k},\boldsymbol{p}}|^2 \,{\rm Re}\left[G^{\rm ret.}_{\boldsymbol{p},\rm R}(t_2-t') G^{<}_{\boldsymbol{k},\rm L}(t'-t_1) + G^{<}_{\boldsymbol{p},\rm R}(t_2-t') G^{\rm adv.}_{\boldsymbol{k},\rm L}(t'-t_1) \right], \tag{10}$$

where $G^{\rm ret.(adv.)}$ is the retarded (advanced) Green's function of a fermion in thermal equilibrium. The lesser component $G^<$ contains the information of the thermal distribution in each reservoir. Here, we take $t_1 = t_2 \equiv t$ and the Fourier transformation

$$I_{\rm qp} = 2\int \frac{d\omega}{2\pi} \sum_{\boldsymbol{p},\boldsymbol{k},\sigma} |t_{\boldsymbol{k},\boldsymbol{p}}|^2 {\rm Re}\left[G^{\rm ret.}_{\boldsymbol{p},\rm R}(\omega) G^{<}_{\boldsymbol{k},\rm L}(\omega) + G^{<}_{\boldsymbol{p},\rm R}(\omega) G^{\rm ret.*}_{\boldsymbol{k},\rm L}(\omega) \right]. \tag{11}$$

Moreover, we use

$$G^{<}_{\boldsymbol{k},\rm j}(\omega) = -2if_{\rm j}(\omega)\,{\rm Im}\,G^{\rm ret.}_{\boldsymbol{k},\rm j}(\omega) \equiv if_{\rm j}(\omega)\mathcal{A}_{\boldsymbol{k},\rm j}(\omega), \tag{12}$$

where

$$f_{\rm j}(\omega) = \frac{1}{\exp(\frac{\omega-\mu_{\rm j}}{T_{\rm j}}) + 1} \tag{13}$$

is the Fermi-Dirac distribution function. We use Matsubara Green's functions in each reservoir reaching thermal equilibrium as a grand-canonical ensemble with $-\mu_{\rm j}\hat{N}_{\rm j}$ and obtain the retarded(advanced) Green's function by the analytic continuation with $\mu_{\rm j}$ as $i\omega_n \to \omega + i\eta - \mu_{\rm j}$ in each reservoir. Then, we obtain

$$I_{\rm qp} = \int \frac{d\omega}{2\pi} \sum_{\boldsymbol{p},\boldsymbol{k},\sigma} |t_{\boldsymbol{k},\boldsymbol{p}}|^2 \mathcal{A}_{\boldsymbol{p},\rm L}(\omega)\mathcal{A}_{\boldsymbol{k},\rm R}(\omega)\left[f_{\rm L}(\omega) - f_{\rm R}(\omega)\right]. \tag{14}$$

Similarly, we obtain the pair current contribution as

$$I_{\rm pair} = 2\sum_{\boldsymbol{q},\boldsymbol{q}'} \int \frac{d\omega}{2\pi} |\omega_{\boldsymbol{q},\boldsymbol{q}'}|^2 \mathcal{B}_{\boldsymbol{q},\rm L}(\omega)\mathcal{B}_{\boldsymbol{q}',\rm R}(\omega)\left[b_{\rm L}(\omega) - b_{\rm R}(\omega)\right], \tag{15}$$

where we used the relation for the two-particle Green's function given $\mathcal{G}^<$ by

$$\mathcal{G}^{<}_{\boldsymbol{q},\rm j}(\omega) = 2ib_{\rm j}(\omega)\,{\rm Im}\,\mathcal{G}^{\rm ret.}_{\boldsymbol{q},\rm j}(\omega) \equiv -ib_{\rm j}(\omega)\mathcal{B}_{\boldsymbol{q},\rm j}(\omega), \tag{16}$$

and the Bose-Einstein distribution function

$$b_{\rm j}(\omega) = \frac{1}{\exp\left(\frac{\omega-\mu_{\rm b,j}}{T_{\rm j}}\right) - 1}, \tag{17}$$

with a bosonic (pair) chemical potential $\mu_{\rm b,j} = 2\mu_{\rm j}$. $\mathcal{G}^{<(>)}$ and $\mathcal{G}^{\rm ret.(adv.)}$ are the lesser (greater) and retarded (advanced) components of two-particle Green's functions, respectively. One can find that $I = I_{\rm qp} + I_{\rm pair}$ obtained from Eqs. (14) and (15) is equivalent to Eq. (4) in the main text. We beriefly note that one may find the correlation between quasiparticle and

tunneling currents in the higher-order contributions such as the term proportional to $t_{\boldsymbol{k},\boldsymbol{p}}^2 w_{\boldsymbol{q},\boldsymbol{q}'}$, which is beyond the scope in this work.

Next, we consider the current noise

$$\mathcal{S} = \frac{1}{2}\int_{-\infty}^{\infty} dt \left(\langle \hat{I}(t)\hat{I}(0)\rangle + \langle \hat{I}(0)\hat{I}(t)\rangle\right). \tag{18}$$

At lowest order of tunneling couplings, we obtain

$$\begin{aligned}\langle \hat{I}(t)\hat{I}(0)\rangle =& \sum_{\boldsymbol{p},\boldsymbol{k},\sigma} |t_{\boldsymbol{p},\boldsymbol{k}}|^2 \left[G^{<}_{\boldsymbol{k},\mathrm{L}}(t)G^{>}_{\boldsymbol{p},\mathrm{R}}(-t) + G^{<}_{\boldsymbol{p},\mathrm{R}}(t)G^{>}_{\boldsymbol{k},\mathrm{L}}(-t)\right] \\ &- 4\sum_{\boldsymbol{q},\boldsymbol{q}'} |w_{\boldsymbol{q},\boldsymbol{q}'}|^2 \left[\mathcal{G}^{<}_{\boldsymbol{q},\mathrm{L}}(t)\mathcal{G}^{>}_{\boldsymbol{q}',\mathrm{R}}(-t) + \mathcal{G}^{<}_{\boldsymbol{q}',\mathrm{R}}(t)\mathcal{G}^{>}_{\boldsymbol{q},\mathrm{L}}(-t)\right],\end{aligned} \tag{19}$$

$$\begin{aligned}\langle \hat{I}(0)\hat{I}(t)\rangle =& \sum_{\boldsymbol{p},\boldsymbol{k},\sigma} |t_{\boldsymbol{p},\boldsymbol{k}}|^2 \left[G^{<}_{\boldsymbol{k},\mathrm{L}}(-t)G^{>}_{\boldsymbol{p},\mathrm{R}}(t) + G^{<}_{\boldsymbol{p},\mathrm{R}}(-t)G^{>}_{\boldsymbol{k},\mathrm{L}}(t)\right] \\ &- 4\sum_{\boldsymbol{q},\boldsymbol{q}'} |w_{\boldsymbol{q},\boldsymbol{q}'}|^2 \left[\mathcal{G}^{<}_{\boldsymbol{q},\mathrm{L}}(-t)\mathcal{G}^{>}_{\boldsymbol{q}',\mathrm{R}}(t) + \mathcal{G}^{<}_{\boldsymbol{q}',\mathrm{R}}(-t)\mathcal{G}^{>}_{\boldsymbol{q},\mathrm{L}}(t)\right].\end{aligned} \tag{20}$$

Collecting them and taking the Fourier transformation, we obtain

$$\mathcal{S} = \mathcal{S}_{\mathrm{qp}} + \mathcal{S}_{\mathrm{pair}}, \tag{21}$$

$$\begin{aligned}\mathcal{S}_{\mathrm{qp}} &= \int_{-\infty}^{\infty}\frac{d\omega}{2\pi}\sum_{\boldsymbol{k},\boldsymbol{p},\sigma} |t_{\boldsymbol{k},\boldsymbol{p},\sigma}|^2 \left[G^{<}_{\boldsymbol{k},\mathrm{L}}(\omega)G^{>}_{\boldsymbol{p},\mathrm{R}}(\omega) + G^{>}_{\boldsymbol{k},\mathrm{L}}(\omega)G^{<}_{\boldsymbol{p},\mathrm{R}}(\omega)\right], \\ \mathcal{S}_{\mathrm{pair}} &= -4\int_{-\infty}^{\infty}\frac{d\omega}{2\pi}\sum_{\boldsymbol{q},\boldsymbol{q}'} |w_{\boldsymbol{q},\boldsymbol{q}'}|^2 \left[\mathcal{G}^{<}_{\boldsymbol{q},\mathrm{L}}(\omega)\mathcal{G}^{>}_{\boldsymbol{q}',\mathrm{R}}(\omega) + \mathcal{G}^{>}_{\boldsymbol{q},\mathrm{L}}(\omega)\mathcal{G}^{<}_{\boldsymbol{q}',\mathrm{R}}(\omega)\right].\end{aligned} \tag{22}$$

Using the relations associated with greater Green's functions

$$G^{>}_{\boldsymbol{p},\mathrm{j}}(\omega) = -i\mathcal{A}_{\boldsymbol{p},\mathrm{j}}(\omega)[1-f_{\mathrm{j}}(\omega)], \quad \mathcal{G}^{>}_{\boldsymbol{q},\mathrm{j}}(\omega) = -i\mathcal{B}_{\boldsymbol{q},\mathrm{j}}(\omega)[1+b_{\mathrm{j}}(\omega)], \tag{23}$$

and the lesser ones given by Eqs. (12) and (16), we obtain

$$\begin{aligned}\mathcal{S}_{\mathrm{qp}} &= \int_{-\infty}^{\infty}\frac{d\omega}{2\pi}\sum_{\boldsymbol{k},\boldsymbol{p},\sigma} |t_{\boldsymbol{k},\boldsymbol{p},\sigma}|^2 \mathcal{A}_{\boldsymbol{k},\mathrm{L}}(\omega)\mathcal{A}_{\boldsymbol{p},\mathrm{R}}(\omega)\left[f_{\mathrm{L}}(\omega)\{1-f_{\mathrm{R}}(\omega)\} + \{1-f_{\mathrm{L}}(\omega)\}f_{\mathrm{R}}(\omega)\right] \\ \mathcal{S}_{\mathrm{pair}} &= 4\int_{-\infty}^{\infty}\frac{d\omega}{2\pi}\sum_{\boldsymbol{q},\boldsymbol{q}'} |w_{\boldsymbol{q},\boldsymbol{q}'}|^2 \mathcal{B}_{\boldsymbol{q},\mathrm{L}}(\omega)\mathcal{B}_{\boldsymbol{q}',\mathrm{R}}(\omega)\left[b_{\mathrm{L}}(\omega)\{1+b_{\mathrm{R}}(\omega)\} + b_{\mathrm{R}}(\omega)\{1+b_{\mathrm{L}}(\omega)\}\right],\end{aligned} \tag{24}$$

which is equivalent to Eq. (6) in the main text. We note that, in Eq. (24), the terms proportional to $[f_{\mathrm{j}}(\omega)\{1-f_{\mathrm{j}}(\omega)\}]$ and $[b_{\mathrm{j}}(\omega)\{1+b_{\mathrm{j}}(\omega)\}]$ ($\mathrm{j}=\mathrm{L},\mathrm{R}$) do not appear in contrast to Ref. (6) because we consider the lowest-order contributions $O(t_{\boldsymbol{k},\boldsymbol{p},\sigma}^2)$ and $O(w_{\boldsymbol{q},\boldsymbol{q}'}^2)$ without the reflection term. Moreover, the correlation of two noises may appear in the higher-order contributions [e.g., $O(t_{\boldsymbol{k},\boldsymbol{p},\sigma}^2 w_{\boldsymbol{q},\boldsymbol{q}'})$], which will be considered in the future work. For a small bias limit at equal temperatures $T_{\mathrm{L}} = T_{\mathrm{R}} \equiv T$ where $\Delta\mu \to 0$ and $f_{\mathrm{R}}(\omega) \to f_{\mathrm{L}}(\omega) \equiv f(\omega)$ with $\mu_{\mathrm{R}} \to \mu_{\mathrm{L}} \equiv \mu$, we obtain

$$f_{\mathrm{L}}(\omega) - f_{\mathrm{R}}(\omega) = -\frac{\partial f(\omega)}{\partial\omega}\Delta\mu + O((\Delta\mu)^2), \tag{25}$$

$$b_{\mathrm{L}}(\omega) - b_{\mathrm{R}}(\omega) = -2\frac{\partial b(\omega)}{\partial\omega}\Delta\mu + O((\Delta\mu)^2). \tag{26}$$

Using

$$f(\omega)\{1-f(\omega)\} = -T\frac{\partial f(\omega)}{\partial\omega}, \quad b(\omega)\{1+b(\omega)\} = -T\frac{\partial b(\omega)}{\partial\omega}, \tag{27}$$

we recover the Onsager's relation

$$\mathcal{S}(\Delta\mu \to 0) = 2T\frac{I}{\Delta\mu}. \tag{28}$$

Moreover, the current and the noise can be rewritten as

$$I_{\rm qp} = \int_{-\infty}^{\infty}\frac{d\omega}{2\pi}\sum_{\boldsymbol{p},\boldsymbol{k},\sigma}|t_{\boldsymbol{k},\boldsymbol{p}}|^2\mathcal{A}_{\boldsymbol{k},{\rm L}}(\omega)\mathcal{A}_{\boldsymbol{p},{\rm R}}(\omega)\left[-\frac{1}{2}\frac{\sinh\left(\frac{\beta_{\rm L}(\omega-\mu_{\rm L})-\beta_{\rm R}(\omega-\mu_{\rm R})}{2}\right)}{\cosh\left(\frac{\beta_{\rm L}(\omega-\mu_{\rm L})}{2}\right)\cosh\left(\frac{\beta_{\rm R}(\omega-\mu_{\rm R})}{2}\right)}\right], \tag{29}$$

$$I_{\rm pair} = 2\int_{-\infty}^{\infty}\frac{d\omega}{2\pi}\sum_{\boldsymbol{q},\boldsymbol{q}'}|w_{\boldsymbol{q},\boldsymbol{q}'}|^2\mathcal{B}_{\boldsymbol{q},{\rm L}}(\omega)\mathcal{B}_{\boldsymbol{q}',{\rm R}}(\omega)\left[-\frac{1}{2}\frac{\sinh\left(\frac{\beta_{\rm b,L}(\omega-\mu_{\rm b,L})-\beta_{\rm R}(\omega-\mu_{\rm b,R})}{2}\right)}{\sinh\left(\frac{\beta_{\rm L}(\omega-\mu_{\rm b,L})}{2}\right)\sinh\left(\frac{\beta_{\rm b,R}(\omega-\mu_{\rm b,R})}{2}\right)}\right], \tag{30}$$

$$\mathcal{S}_{\rm qp} = \int_{-\infty}^{\infty}\frac{d\omega}{2\pi}\sum_{\boldsymbol{k},\boldsymbol{p},\sigma}|t_{\boldsymbol{k},\boldsymbol{p}}|^2\mathcal{A}_{\boldsymbol{k},{\rm L}}(\omega)\mathcal{A}_{\boldsymbol{p},{\rm R}}(\omega)\left[-\frac{1}{2}\frac{\cosh\left(\frac{\beta_{\rm L}(\omega-\mu_{\rm L})-\beta_{\rm R}(\omega-\mu_{\rm R})}{2}\right)}{\cosh\left(\frac{\beta_{\rm L}(\omega-\mu_{\rm L})}{2}\right)\cosh\left(\frac{\beta_{\rm R}(\omega-\mu_{\rm R})}{2}\right)}\right], \tag{31}$$

$$\mathcal{S}_{\rm pair} = 4\int_{-\infty}^{\infty}\frac{d\omega}{2\pi}\sum_{\boldsymbol{q},\boldsymbol{q}'}|w_{\boldsymbol{q},\boldsymbol{q}'}|^2\mathcal{B}_{\boldsymbol{q},{\rm L}}(\omega)\mathcal{B}_{\boldsymbol{q}',{\rm R}}(\omega)\left[-\frac{1}{2}\frac{\cosh\left(\frac{\beta_{\rm L}(\omega-\mu_{\rm b,L})-\beta_{\rm R}(\omega-\mu_{\rm b,R})}{2}\right)}{\sinh\left(\frac{\beta_{\rm L}(\omega-\mu_{\rm b,L})}{2}\right)\sinh\left(\frac{\beta_{\rm R}(\omega-\mu_{\rm b,R})}{2}\right)}\right]. \tag{32}$$

In particular, considering the large-biased limit where

$$\tanh\left(\frac{\beta_{\rm L}(\omega-\mu_{\rm L})-\beta_{\rm R}(\omega-\mu_{\rm R})}{2}\right)\simeq\tanh\left(\frac{\beta_{\rm L}(\omega-\mu_{\rm b,L})-\beta_{\rm R}(\omega-\mu_{\rm b,R})}{2}\right)\simeq 1, \tag{33}$$

is satisfied, we obtain

$$\mathcal{S}_{\rm qp}(\Delta\mu\to\infty)\to I_{\rm qp}, \qquad \mathcal{S}_{\rm pair}(\Delta\mu\to\infty)\to 2I_{\rm pair}, \tag{34}$$

where we have denoted $I\equiv I_{\rm qp}+I_{\rm pair}$. The result of Eq. (34) motivates us to consider the Fano factor

$$F=\frac{\mathcal{S}}{I}=\frac{\mathcal{S}_{\rm qp}+\mathcal{S}_{\rm pair}}{I_{\rm qp}+I_{\rm pair}}. \tag{35}$$

Then, one can see that the Fano factor F in a large-biased junction changes from 1 to 2 reflecting the ratio between $I_{\rm qp}$ and $I_{\rm pair}$.

Many-body T-matrix approximation

To demonstrate this, we employ the many-body TMA to calculate spectral functions $\mathcal{A}_{\boldsymbol{k},{\rm j}}(\omega)$, $\mathcal{B}_{\boldsymbol{q},{\rm j}}(\omega)$, and $\mu_{\rm j}$ for given densities $N_{\rm j}$ in the BCS–BEC crossover regime (3). The single-particle propagator is given by

$$G_{\boldsymbol{k},{\rm j}}(i\omega_n)=\frac{1}{G^0_{\boldsymbol{k},{\rm j}}(i\omega_n)^{-1}-\Sigma_{\boldsymbol{k},{\rm j}}(i\omega_n)}, \tag{36}$$

$$\Sigma_{\boldsymbol{k},{\rm j}}(i\omega_n)=T_{\rm j}\sum_{\boldsymbol{q},\ell}\Gamma_{\boldsymbol{q},{\rm j}}(i\nu_\ell)G^0_{\boldsymbol{q}-\boldsymbol{k},{\rm j}}(i\nu_\ell-i\omega_n), \tag{37}$$

where $G^0_{\boldsymbol{k},{\rm j}}(i\omega_n)=(i\omega_n-\xi_{\boldsymbol{k},{\rm j}})^{-1}$ denotes the bare propagator and $\Sigma_{\boldsymbol{k},{\rm j}}(i\omega_n)$ denotes the TMA self-energy. Following a standard TMA procedure (7), the T-matrix $\Gamma_{\boldsymbol{q},{\rm j}}(i\nu_\ell)$ is formulated by incorporating the particle–particle multiple scattering as

$$\Gamma_{\boldsymbol{q},{\rm j}}(i\nu_\ell)=g\left[1-g\Pi_{\boldsymbol{q},{\rm j}}(i\nu_\ell)\right]^{-1}, \tag{38}$$

using the bare two-body propagator given as

$$\Pi_{\boldsymbol{q},{\rm j}}(i\nu_\ell)=-T_{\rm j}\sum_{\boldsymbol{p},n}G^0_{\boldsymbol{p}+\boldsymbol{q}/2,{\rm j}}(i\omega_n+i\nu_\ell)G^0_{-\boldsymbol{p}+\boldsymbol{q}/2,{\rm j}}(-i\omega_n). \tag{39}$$

The fermion (boson) Matsubara frequency is denoted by ω_n (ν_ℓ). Furthermore, we define the dressed two-body propagator (8) as

$$\mathcal{G}_{\boldsymbol{q},{\rm j}}(i\nu_\ell)=\Pi_{\boldsymbol{q},{\rm j}}(i\nu_\ell)\left[1+\Pi_{\boldsymbol{q},{\rm j}}(i\nu_\ell)\Gamma_{\boldsymbol{q},{\rm j}}(i\nu_\ell)\right]. \tag{40}$$

The spectral functions can be obtained from the analytic continuation as $\mathcal{A}_{\boldsymbol{k},{\rm j}}(\omega)=-2\,{\rm Im}\,G_{\boldsymbol{k},{\rm j}}(i\omega_n\to\omega-\mu_{\rm j}+i\eta)$ and $\mathcal{B}_{\boldsymbol{q},{\rm j}}(\omega)=-2\,{\rm Im}\,\mathcal{G}_{\boldsymbol{q},{\rm j}}(i\nu_\ell\to\omega-\mu_{\rm b,j}+i\eta)$ with an infinitesimal small number η.

Retarded propagators in the dilute reservoir

For the single-particle Green's function in the reservoir R at dilute limit, we employ the non-interacting one given by

$$G^{\text{ret.}}_{\boldsymbol{p},\text{R}}(\omega) = \frac{1}{\omega + i\eta - \epsilon_{\boldsymbol{p}}}, \tag{41}$$

where the self-energy correction is ignored [noting $\epsilon_{\boldsymbol{p}} = p^2/(2m)$]. For the two-body sector, we can rewrite the lowest-order two-body propagator as

$$\Pi^{\text{ret.}}_{\boldsymbol{q},\text{j}}(\omega) \equiv \Pi_{\boldsymbol{q},0}(\omega) + \Xi_{\boldsymbol{q},\text{j}}(\omega), \tag{42}$$

where

$$\Pi_{\boldsymbol{q},0}(\omega) = \sum_{\boldsymbol{p}} \frac{1}{\omega + i\eta - \epsilon_{\boldsymbol{p}+\boldsymbol{q}/2} - \epsilon_{-\boldsymbol{p}+\boldsymbol{q}/2}} \tag{43}$$

and

$$\Xi_{\boldsymbol{q},\text{j}}(\omega) = -\sum_{\boldsymbol{p}} \frac{f_{\text{j}}(\epsilon_{\boldsymbol{p}+\boldsymbol{q}/2}) + f_{\text{j}}(\epsilon_{-\boldsymbol{p}+\boldsymbol{q}/2})}{\omega + i\eta - \epsilon_{\boldsymbol{p}+\boldsymbol{q}/2} - \epsilon_{-\boldsymbol{p}+\boldsymbol{q}/2}} \tag{44}$$

are the in-vacuum two-body Green's function and the medium correction, respectively (for more details, see e.g., Refs. (3, 9)). Taking $\alpha^2 = q^2/4 - m\omega - i\delta$, we can analytically obtain

$$\Pi_{\boldsymbol{q},0}(\omega) = -\frac{m\Lambda}{2\pi^2} + \frac{m\alpha}{2\pi^2}\tan^{-1}\left(\frac{\Lambda}{\alpha}\right), \tag{45}$$

where Λ is an ultraviolet cutoff. Note that Λ is renormalized via

$$\frac{m}{4\pi a} = \frac{1}{g} + \frac{m\Lambda}{2\pi^2}, \tag{46}$$

which leads to

$$\begin{aligned} \frac{1}{\Gamma^{\text{ret.}}_{\boldsymbol{q},\text{j}}(\omega)} &= \frac{m}{4\pi a} - \Pi^{\text{ret.}}_{\boldsymbol{q},\text{j}}(\omega) - \frac{m\Lambda}{2\pi^2} \\ &\simeq \frac{m}{4\pi a} - \Xi_{\boldsymbol{q}}(\omega) - \frac{m\alpha}{4\pi} \end{aligned} \tag{47}$$

where the ultraviolet divergence is cancelled ($\tan^{-1}\left(\frac{\Lambda}{\alpha}\right) \simeq \pi/2$ is used in the second line).

In the dilute limit, the fermionic medium correction $\Xi_{\boldsymbol{q},\text{R}}(\omega)$ is negligible. In this case, one can approximately obtain

$$\mathcal{G}^{\text{ret.}}_{\boldsymbol{q},\text{R}}(\omega) \simeq \Pi_{\boldsymbol{q},0}(\omega)\left[1 - g\Pi_{\boldsymbol{q},0}(\omega)\right]^{-1}. \tag{48}$$

where $\mathcal{G}^{\text{ret.}}_{\boldsymbol{q},\text{R}}(\omega)$ does not involve any poles on the real frequency axis (i.e. bound states) at $a^{-1} < 0$. Note that the two-body continuum exists above $\omega = q^2/(4m)$. In the weak-coupling side ($a < 0$), we obtain

$$\mathcal{B}_{\boldsymbol{q},\text{R}}(\omega) = -2\,\text{Im}\,\mathcal{G}^{\text{ret.}}_{\boldsymbol{q},\text{R}}(\omega) = 0. \quad (\omega < q^2/4m). \tag{49}$$

Simultaneously, the frequency integration is restricted as $\omega > 0$. This fact indicates that particles in the reservoir L are transferred to the two-body continuum in the reservoir R via the two-body tunneling process in the weak-coupling side ($a < 0$). On the other hand, in the strong-coupling limit ($a \to +\infty$), we obtain (8, 10)

$$\mathcal{G}^{\text{ret.}}_{\boldsymbol{q},\text{R}}(\omega) \simeq \left(\frac{m\Lambda}{2\pi^2}\right)^2 \frac{8\pi}{m^2 a} \frac{1}{\omega + i\eta - \frac{q^2}{4m} + E_{\text{b}}} \quad (\Lambda \to \infty), \tag{50}$$

which is proportional to the bosonic Green's function of a bound molecule with the binding energy $E_{\text{b}} = 1/(ma^2)$. Thus, in the strong-coupling regime ($a > 0$), particles in the reservoir L can be transferred to the molecular bound states in the reservoir R via the two-body tunneling process.

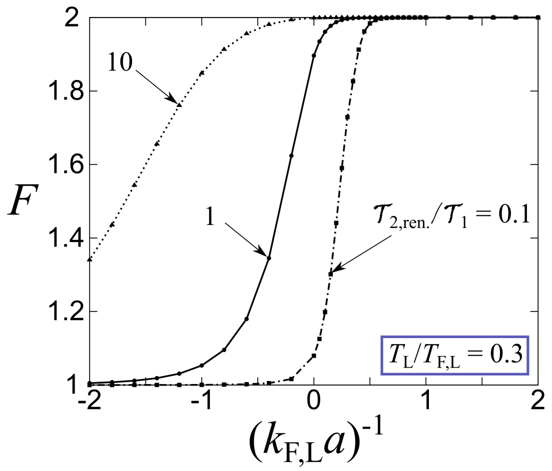

Fig. S2. Fano factor F throughout the BCS-BEC crossover at different tunneling-coupling ratio $\mathcal{T}_{2,\mathrm{ren.}}/\mathcal{T}_1$. The temperature is taken as $T_\mathrm{L}/T_\mathrm{F,L} = 0.3$. One can see that F changes from 1 to 2 with increasing the interaction strength regardless of different $\mathcal{T}_{2,\mathrm{ren.}}/\mathcal{T}_1$.

Large-bias limit

In the main text, we considered a situation where fermions in the strongly-correlated reservoir L with a finite density N_L go through the tunneling junction to the dilute reservoir R with a vanishing density $N_\mathrm{R} \to 0$, i.e., $\mu_\mathrm{R} \to -\infty$. While we take the same temperatures $T_\mathrm{L} = T_\mathrm{R}$ in the two reservoirs, T_R does not affect the result in the present case of $\mu_\mathrm{R} \to -\infty$ because the fugacity $z_\mathrm{R} = e^{\mu_\mathrm{R}/T_\mathrm{R}}$ characterizing the distribution vanishes regardless of the value of T_R. The condition of the large bias limit [$\mu_\mathrm{R} = T_\mathrm{R}\ln(z_\mathrm{R}) \to -\infty$] is unchanged in both BCS and BEC sides at nonzero temperatures because the dilute reservoir R obeys the Boltzmann statistics. Indeed, we obtain vanishing N_R as (11)

$$N_\mathrm{R} = 2z_\mathrm{R}\left(\frac{2\pi}{mT_\mathrm{R}}\right)^{\frac{3}{2}} + O(z_\mathrm{R}^2) \to 0 \quad (z_\mathrm{R} \to 0). \qquad [51]$$

The number density N_L of the L-reservoir can be numerically obtained from

$$N_\mathrm{L} = T_\mathrm{L}\sum_{\boldsymbol{p},\sigma,n} G_{\boldsymbol{p},\mathrm{L}}(i\omega_n). \qquad [52]$$

In this regard, we normalize physical quantities by using the Fermi energy $E_\mathrm{F,L} = (3\pi^2 N_\mathrm{L})^{\frac{2}{3}}/(2m)$ and momentum $k_\mathrm{F,L} = (3\pi^2 N_\mathrm{L})^{\frac{1}{3}}$.

While the Fano factor is well described by the Onsager's relation $F^{-1}(\Delta\mu \to 0) = \frac{\Delta\mu}{2T}$ in the low-bias regime, F^{-1} approaches the large-bias limit ($\mu_\mathrm{R} \to -\infty$) when $\Delta\mu/E_\mathrm{F,L} \gtrsim 1$. This indicates that it is sufficient to reach the large-bias limit when $\Delta\mu$ is larger than the many-body scale of the reservoir, that is, $E_\mathrm{F,L}$. We note that $\Delta\mu$ can be controllable in cold atomic experiments by preparing the reservoirs with the large density imbalance.

1. Different tunneling-coupling ratio

Figure S2 shows the calculated Fano factor F with different tunneling-coupling ratio $\mathcal{T}_{2,\mathrm{ren.}}/\mathcal{T}_1$ in the entire BCS-BEC crossover regime at $T_\mathrm{L}/T_\mathrm{F,L} = 0.3$. While in the main text we employed $\mathcal{T}_{2,\mathrm{ren.}}/\mathcal{T}_1 = 1$, this ratio depends on the actual detailed setups in each experiment. If the two-body tunneling is relatively strong as $\mathcal{T}_{2,\mathrm{ren.}}/\mathcal{T}_1 = 10$, F is close to 2 even in the weak-coupling side [$(k_\mathrm{F,L}a)^{-1} \simeq -1$]. However, F decreases at weaker coupling even in this case. On the other hand, in the case with $\mathcal{T}_{2,\mathrm{ren.}}/\mathcal{T}_1 = 0.1$, F remains to be close to 1 even around unitarity. Nevertheless, F rapidly increases around $(k_\mathrm{F,L}a)^{-1} = 0.3$ and consequently reaches $F = 2$ in the strong-coupling limit.

In this way, the detailed structure of the tunneling junction affects how F increases in the BCS-BEC crossover regime. However, our conclusion that $F = 1$ and $F = 2$ are achieved in the BCS and BEC limits, respectively, is unchanged even for different tunneling-coupling ratios. In other words, the pair tunneling process inevitably occurs in the strong-coupling regime even for an infinitesimally small pair-tunneling coupling $\mathcal{T}_2$. This is a natural consequence in the sense that the system is dominated by bound molecules and hence there are no single-particle states in such a regime.

We note that the value of $\mathcal{T}_{2,\mathrm{ren.}}/\mathcal{T}_1$ is associated with the potential barrier and the interaction strength (1). While it is not so straightforward to estimate $\mathcal{T}_{2,\mathrm{ren.}}/\mathcal{T}_1$ in each experimental setup, it is sufficient to observe F at the regime where the anomalously large tunneling current can be found [e.g., at unitarity observed in Ref. (12)] for our purpose of detecting the pair-tunneling current.

References

1. H Tajima, D Oue, M Matsuo, Multiparticle tunneling transport at strongly correlated interfaces. *Phys. Rev. A* **106**, 033310 (2022).
2. DJ Griffiths, DF Schroeter, *Introduction to quantum mechanics.* (Cambridge university press), (2018).
3. Y Ohashi, H Tajima, P van Wyk, Bcs-bec crossover in cold atomic and in nuclear systems. *Prog. Part. Nucl. Phys.* **111**, 103739 (2020).
4. C Chin, R Grimm, P Julienne, E Tiesinga, Feshbach resonances in ultracold gases. *Rev. Mod. Phys.* **82**, 1225–1286 (2010).
5. Y Sagi, TE Drake, R Paudel, DS Jin, Measurement of the homogeneous contact of a unitary fermi gas. *Phys. Rev. Lett.* **109**, 220402 (2012).
6. YM Blanter, M Büttiker, Shot noise in mesoscopic conductors. *Phys. reports* **336**, 1–166 (2000).
7. W Zwerger, *The BCS-BEC crossover and the unitary Fermi gas.* (Springer Science & Business Media) Vol. 836, (2011).
8. P Pieri, GC Strinati, Strong-coupling limit in the evolution from bcs superconductivity to bose-einstein condensation. *Phys. Rev. B* **61**, 15370–15381 (2000).
9. GC Strinati, P Pieri, G Röpke, P Schuck, M Urban, The bcs–bec crossover: From ultra-cold fermi gases to nuclear systems. *Phys. Reports* **738**, 1–76 (2018) The BCS–BEC crossover: From ultra-cold Fermi gases to nuclear systems.
10. N Andrenacci, P Pieri, GC Strinati, Evolution from bcs superconductivity to bose-einstein condensation: Current correlation function in the broken-symmetry phase. *Phys. Rev. B* **68**, 144507 (2003).
11. V Ngampruetikorn, MM Parish, J Levinsen, High-temperature limit of the resonant fermi gas. *Phys. Rev. A* **91**, 013606 (2015).
12. G Del Pace, WJ Kwon, M Zaccanti, G Roati, F Scazza, Tunneling transport of unitary fermions across the superfluid transition. *Phys. Rev. Lett.* **126**, 055301 (2021).
